# The Protective Effects of Metformin and Vitamin C and Their Co-Administration in Bleomycin-Induced Pulmonary Fibrosis in Mice

**DOI:** 10.1155/adpp/5227142

**Published:** 2025-04-06

**Authors:** Mohammad Ebrahim Abbaszadeh, Mohammad Rafi Khezri, Morteza Ghasemnejad-Berenji

**Affiliations:** ^1^Department of Anatomical Sciences, Faculty of Medicine, Urmia University of Medical Sciences, Urmia, Iran; ^2^Urmia University of Medical Sciences, Urmia, Iran; ^3^Department of Pharmacology and Toxicology, School of Pharmacy, Urmia University of Medical Sciences, Urmia, Iran; ^4^Research Center for Experimental and Applied Pharmaceutical Sciences, Urmia University of Medical Sciences, Urmia, Iran

**Keywords:** ascorbic acid, bleomycin, fibrosis, lung, metformin

## Abstract

Bleomycin, an antibacterial antibiotic, is used in chemotherapy and is effective against various forms of human carcinomas. However, its use is limited due to its tendency to cause pulmonary fibrosis. Oxidative stress and excessive expression of TGF beta occur in pulmonary fibrosis, leading to cellular death, inflammation, and additional damage to lung tissue. Metformin has the ability to reduce oxidative stress and lower the level of TGF beta by activating AMPK. Additionally, ascorbic acid possesses potent antioxidant characteristics. Consequently, we decided to investigate the effects of these two medications on pulmonary fibrosis and compare with methyl prednisolone. Thirty-six adult mice were categorized into 6 distinct groups: Control, bleomycin (bleo), bleo + methyl prednisolone, bleo + metformin, bleo + ascorbic acid, bleo + metformin + ascorbic acid. Pulmonary fibrosis was induced by the administration of bleomycin in all groups, except for the control group. Subsequently, medications were administered for a duration of 14 days. Ultimately, the mice were sacrificed and lung tissues were obtained for biochemical and histological examination. As shown by biochemical and histological analysis, all treatment groups showed a decrease in oxidative stress factors, inflammation, and lung tissue fibrosis; however, the effects of administering metformin and ascorbic acid together were noticeable. Our study found that administering metformin and ascorbic acid over a period of 14 days, either alone or in combination, may contribute to the repair of pulmonary fibrosis. However, our data indicate that the combined therapy of these drugs provided a better result.

## 1. Introduction

Bleomycin is an antibacterial antibiotic utilized in chemotherapy, which has demonstrated efficacy against several types of human carcinomas. This drug has the lowest level of toxicity against hematological tissues and the immune system. Due of its distinct toxicity profile, it can be administered alongside other chemotherapeutic medicines. Regrettably, the practical utilization of this medicine is limited due to complications arising from pulmonary fibrosis [1]. Bleomycin is frequently used in animal models to produce pulmonary fibrosis. Its administration causes distinct inflammatory responses and fibrosis in the lungs [2].

Oxidative stress, resulting from the presence of reactive oxygen and nitrogen species and the peroxidation of membrane lipids, is a contributing factor to the progression of pulmonary fibrosis. Oxidative stress induces programmed cell death in alveolar epithelial cells and leads to the degradation of the basement membrane. It also limits the formation of lung epithelial tissue and inhibits the process of lung repair and the exchange of breathing gases [3].

TGF beta is overexpressed in cases of pulmonary fibrosis. TGF beta stimulates the excessive production and accumulation of extracellular matrix proteins such as fibronectin and type 1 collagen in the lungs. This is achieved by promoting the transformation of epithelial cells into mesenchymal tissue and fibroblasts into myofibroblasts [4]. Furthermore, TGF beta stimulates the production of reactive oxygen species and facilitates the proliferation of inflammatory cells [5]. Tumor necrosis factor alpha (TNF alpha) and interleukin-6 (IL-6), which are secreted by activated T cells and macrophages, have been shown to have a role in both inflammation and the regeneration of lung tissue after damage [6].

Recent research has demonstrated that malfunctioning of AMPK is a significant factor in the progression of pulmonary fibrosis produced by bleomycin [7]. Research has shown that the activation of AMPK decreases the occurrence of TGF beta-induced fibrogenesis in hepatic stellate cells. Consequently, it has been determined that the activation of AMPK could serve as an innovative therapeutic approach for the treatment of liver fibrosis [8].

Metformin is a pharmacological agent used to treat diabetes by activating AMPK. In mice with asthma, it has been observed that there is a reduction in bronchial fibrosis, thickness of the smooth muscle layer, and mucus production. These effects are associated with a decrease in oxidative stress and an increase in AMPK activation [9]. Metformin prevents the development of fibrosis generated by gefitinib in human fetal fibroblasts by two mechanisms: firstly, by obstructing the signaling of TGF- and IL-6 and reducing their production; and secondly, by reversing the process of extracellular matrix formation [10]. In addition, it actively inhibits reactive oxygen species, decreases lipid peroxidation, diminishes carbonyl protein, enhances the antioxidant system, and ultimately reduces oxidative stress levels in diabetes mellitus [11–13].

Vitamin C or ascorbic acid is a powerful antioxidant that aids several cellular activities in both the innate and acquired immune systems. This substance creates a protective layer in the skin that prevents infections and helps remove harmful substances. As a result, it shields the skin from damage caused by environmental oxidative stress. Ascorbic acid rapidly donates electrons to counteract the harm produced by oxidant biomolecules [14]. Additionally, it serves as a cofactor for the lysyl and prolyl hydroxylase enzymes, which are essential for stabilizing collagen and facilitating the transportation of fatty acids to the mitochondria for the production of metabolic energy [15, 16]. In addition, ascorbic acid has the potential to decrease the levels of IL-6 and TGF-β in lung fibrosis generated by paraquat. It can also diminish the presence of neutrophils, macrophages, and lymphocytes in the fluid obtained from bronchoalveolar lavage [17].

In this study, we examined the protective effects of metformin and vitamin C, both individually and in combination, on bleomycin-induced lung fibrosis in mice. We also compared these effects to those of prednisolone, an approved corticosteroid drug for pulmonary fibrosis.

## 2. Methods and Materials

### 2.1. Animals and Experimental Designs

A total of 36 adult albino male mice, with weights ranging from 20 to 30 g, were obtained from the Department of Pharmacology at Urmia University of Medical Sciences. They were kept in a controlled setting with a 12 h light and 12 h dark cycle, and were provided with unlimited access to food and water. The temperature was maintained at a constant range of 21°C–22°C.

The mice were divided into six groups using a random method, with six animals in each group (*n* = six animals/group): Group 1: Control group, mice were not subjected to any interventions. Group 2: Bleomycin group, this group involved the induction of pulmonary fibrosis in mice by administering a single dosage of bleomycin (0.06 mg/animal) [18] directly into their trachea. Group 3 consisted of mice that were administered metformin (100 mg/kg, i.p.) [19] in addition to bleomycin for a duration of 14 days. Group 4 consisted of mice that were administered both bleomycin and vitamin C (150 mg/kg, i.p.) [17] for a duration of 14 days. Group 5 consisted of mice that were administered methylprednisolone (4 mg/kg, i.p.) in addition to bleomycin for a duration of 14 days. Group 6 consisted of mice that were administered vitamin C and metformin, along with bleomycin, at specified doses for a duration of 14 days.

Following the administration of the final medicine dose, the weights of the mice were assessed. Subsequently, the mice were sedated using xylazine/ketamine (10/90 mg/kg, i.p.) in a sterile environment after a period of 24 h. Following the separation of the chest, the lungs were extracted for the purpose of measuring their weight and conducting biochemical and histological investigations.

The right lungs were fixed in 10% phosphate-buffered formalin for hematoxylin-eosin (H&E) and Mason trichrome (MT) staining. The left lungs were immediately fixed at −80 centigrade for analysis of alterations in tissue superoxide dismutase (SOD), catalase (CAT), glutathione peroxidase (GPx), and malondialdehyde (MDA) levels.

### 2.2. Biochemical Analysis

The lungs were thawed at a temperature of −80°C and subsequently extracted for biochemical examination. To evaluate biochemical variables, a 10% tissue homogenate was prepared using a 0.1 M phosphate buffer with a pH of 7.4. The level of lipid peroxidation in the cell membrane is measured by the presence of MDA in tissues. The MDA levels in this study were measured using a spectrophotometric approach, following the methodology reported by Ohkawa et al. [20]. The Winterbourn et al. [21] method was employed to assess the efficacy of SOD in inhibiting the reduction of Nitro blue tetrazolium by superoxide ions, hence measuring SOD enzyme activity. The Aebi [22] method was employed to assess the CAT enzyme's activity by measuring the breakdown of hydrogen peroxide (H_2_O_2_) at a wavelength of 240 nm. The assessment of GPx activity in lung tissue was conducted using a spectrophotometric method at a temperature of 37 degrees Celsius and a wavelength of 340 nm, following the methods proposed by Paglia and Valentine [23].

### 2.3. Histopathological Analysis

The lung samples were immersed in a 10% solution of formalin buffered with phosphate for 48 h to facilitate histological examination. Subsequently, they were extracted and encased in paraffin. Tissue samples were sliced into sections that were 5 μm thick. These sections were then mounted on slides and treated with H&E and MT stains. The slides were scrutinized under a light microscope with a magnification of 100x. The degree of tissue injury and inflammation was assessed using H&E staining, whereas the degree of fibrosis was evaluated using MT staining.

The injury's severity was assessed through scoring, and the ratings of each group were subsequently compared. The scoring methodology is outlined as follows:

Inflammatory infiltration with no inflammation (score 0); the bronchi and vessels exhibited local inflammation, with a thin layer of inflammatory cells (1–5 cells thick) surrounding most of them (score 2). Additionally, a thick layer of inflammatory cells (more than 5 cells thick) surrounded most of the bronchi and vessels (score 3). Complete inflammation was observed around the bronchi and vessels (score 4) [24].

Pulmonary fibrosis is assessed using the Ashcroft scoring method, which takes into account the presence of normal lung tissue (score 0). The minimal thickness of lung fibrosis in the walls of alveolar and bronchiolar arteries is one unit (score 1). The average thickness of undamaged lung walls (scoring 2-3); increased fibrosis with specific damage to lung structure and the formation of fibrosis clusters or tiny masses (score 5-4); the lung structure had significant damage and exhibited extensive fibrosis, resulting in the formation of honeycomb lung (scoring 7-6) and full obliteration of the lung field (score 8) [25].

### 2.4. Statistical Analysis

The findings were reported as the average value plus or minus the standard deviation. The data was analyzed utilizing the GraphPad Prism software and the one-way ANOVA statistical test. Subsequently, Tukey's post hoc test was employed to statistically compare the results obtained from different groups. A significant level was determined as a probability (P) value less than 0.05.

## 3. Results

### 3.1. Biochemical Analyses

#### 3.1.1. CAT

**Figure 1:**
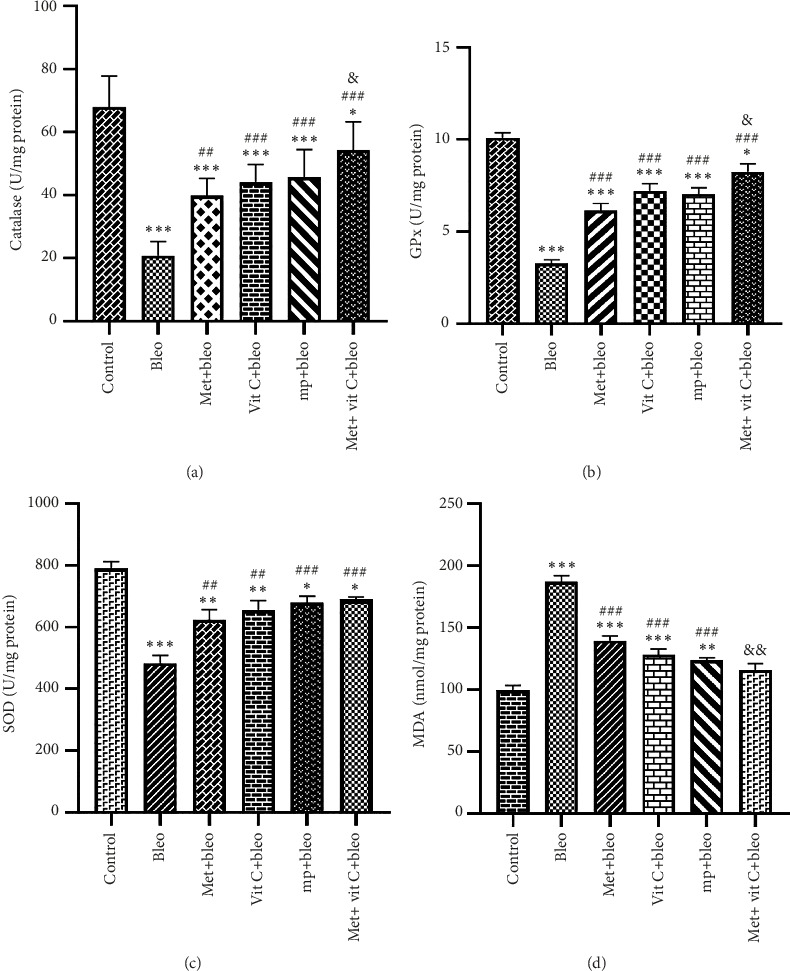
Effect of metformin and ascorbic acid on oxidative stress parameters in different experimental groups. (a) Catalase, (b) GPx, (c) SOD activity, and (d) MDA levels of mice lung tissue in all groups. ^∗, ∗∗, ∗∗∗^: significant difference with control group, *p* < 0.05, *p* < 0.01, and *p* < 0.001, respectively. ^#, ##, ###^: significant difference with bleomycin group, *p* < 0.05, *p* < 0.01, and *p* < 0.001, respectively. ^&, &&^: significant difference with bleomycin + metformin group, *p* < 0.05 and *p* < 0.01, respectively.

Figure 1(a) illustrates the variations in the activity of CAT among various groups. The control group had the highest level of CAT activity, which experienced a significant drop following the administration of bleomycin (*p* < 0.001). Nevertheless, the levels of CAT activity in the metformin (met) (*p* < 0.01), vitamin C (vit C), methylprednisolone (mp), and met + vit C (*p* < 0.001) treatment groups exhibited a considerable increase as compared to the bleomycin (bleo) group. The rise in question was particularly noticeable in the group that received both metformin and vitamin C, resulting in a significant difference compared to the group that only received metformin (*p* < 0.05).

#### 3.1.2. GPx

The bleo group had the lowest level of GPx enzyme activity among the groups examined, and this difference was statistically significant compared to the control group (*p* < 0.001). Nevertheless, the enzyme's activity in the therapy group under investigation was markedly greater than that in the bleo group (*p* < 0.001). Conversely, within the groups receiving therapy, the met + vit C group exhibited the highest level of GPx activity. This group showed a significant difference compared to the metformin group (*p* < 0.05) (Figure 1(b)).

#### 3.1.3. SOD

Our research indicates that the control group had the highest level of SOD enzyme activity compared to the other groups under study. However, following the administration of bleomycin, this level saw a substantial drop (*p* < 0.001). Nevertheless, the therapeutic methods employed in this investigation resulted in a significant elevation in SOD activity following the administration of bleomycin (*p* < 0.05). Notably, the met + vit c group exhibited the most substantial rise. Furthermore, there was no notable disparity observed in the SOD activity across the treatment groups (*p* > 0.05) (Figure 1(c)).

#### 3.1.4. MDA

The data revealed a considerable increase in MDA activity following the administration of bleomycin, which was markedly different from the control group (*p* < 0.001). Nevertheless, the therapeutic methods employed in this investigation resulted in a substantial reduction in MDA level when compared to the bleo group (*p* < 0.001). This reduction was most pronounced in the met + vit C group and exhibited significant difference with the metformin group (*p* < 0.01) (Figure 1(d)).

### 3.2. Histopathological Evaluation

**Figure 2:**
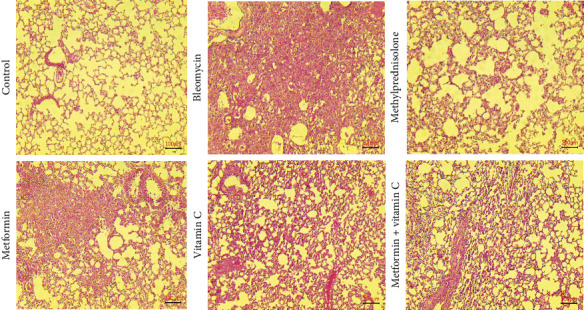
H&E staining of mice lung tissue in all groups. Inflammatory cells and inflammation increased at lung tissue after bleomycin administration but this inflammation decreased following administration of metformin, ascorbic acid, methyl prednisolone and met + ascorbic acid, however it was more noticeable in met + ascorbic acid and methyl prednisolone administration.

Histological analysis using H&E staining revealed that intratracheal treatment of bleomycin induced significant inflammation, disruption of alveolar architecture, substantial thickening of the septum, and interstitial infiltration, as compared to the control group. Nevertheless, the therapy groups exhibited a decrease in inflammation and inflammatory cells, as well as a prevention of alveoli shrinkage and septum thickening. The effects were most pronounced when metformin and ascorbic acid were used together, and these effects were not substantially different from those observed in the methylprednisolone group (Figure 2 and Table 1).

**Figure 3:**
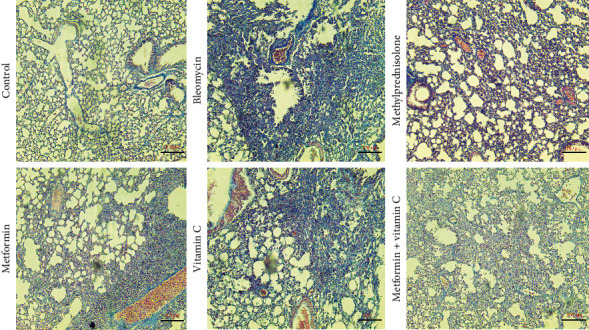
Masson trichrome staining of mice lung tissue in all groups. Bleomycin administration increased collagen fibers (blue area) showing lung tissue fibrosis. Pulmonary fibrosis decreased following administration of metformin, ascorbic acid, methyl prednisolone and met + ascorbic acid, however it was more noticeable in met + ascorbic acid and methyl prednisolone administration.

Furthermore, Masson's trichrome staining revealed a substantial increase in collagen accumulation and notable fibrosis in the lung tissue of the group that received intratracheal bleomycin, as compared to the control group. Nevertheless, during the assessment of the therapy groups, it was noted that the metformin and ascorbic acid treatment group had a more severe decrease in collagen deposition, intensity of inflammation, and degree of fibrosis compared to the other groups (Figure 3 and Table 1).

## 4. Discussion

The main purpose of this study was to evaluate the protective effects of metformin alone or in combination with ascorbic acid on pulmonary fibrosis. The results of our study significantly showed that metformin and ascorbic acid combination therapy abrogated the tissue histopathologic changes induced by bleomycin. Indeed, biochemical assays showed that metformin combined with ascorbic acid enhanced antioxidative response to ROS generated by bleomycin in the lungs.

Pulmonary fibrosis is a class of idiopathic interstitial pneumonia with a high-rate mortality and life expectancy of 2-3 years after diagnosis. There are a wide variety of symptoms for IPF, the most important of which are hypoxemia, dyspnea, prominent lung infiltrates on radiographs, and recruitment of fibroblasts in lung tissue, that are resulted by the scarring of lung tissue following pulmonary fibrosis [26, 27]. Whereas the exact molecular mechanisms of fibrosis in pulmonary fibrosis are not fully known yet, there is a general phenomenon in its pathogenesis showing genetic predisposition to subclinical injuries into the epithelium of alveoli, that is, resulted by pulmonary failure of cyst re-epithelialization [28]. Activated immune cells within the alveoli generate large magnitudes of growth factors and cytokines which enhance pulmonary fibroblast activation, proliferation, and differentiation into myofibroblasts, leading to excessive accumulation of collagen and their progression into the lung parenchyma, resulting in scars and loss of function [29]. Prevention of lung fibrosis induced by drugs and chemicals is a significant unmet medical requirement which is growing in importance, most importantly during cancer treatment procedure, however, supportive therapeutic interventions must be well‐tolerated. Bleomycin is a chemotherapeutic drug used to treat a wide variety of cancers including ovarian cancer, head and neck squamous cell carcinoma, testicular carcinomas, and lymphoma [30]. However, one of the main complications of Bleomycin is oxidative injury of lungs that limits its usage in clinical setting. Increased generation of ROS, that leads to oxidative stress, has been introduced as one of the main molecular mechanisms of pulmonary fibrosis induced by bleomycin. In order to produce excessive magnitudes of ROS, formation of a complex consist of bleomycin, molecular oxygen and divalent ions such as iron is necessary, that subsequently leads to form free radical species such as hydroxyl radicals and O_2_^−^. During the next step, generated complex binds to macromolecules such as DNA helix, leading to DNA strand breaks followed by lipid peroxidation and apoptosis induction [31, 32]. On the other hand, high amounts of ROS contributes disturbs the ability of cellular antioxidative to combat oxidative damage. Numerous studies have indicated that bleomycin administration alters the activity of main antioxidative parameters in the lung, such as SOD, CAT, and GPx [33, 34]. The results of our study is clearly in agreement with these findings in a way that we observed a significant alteration in oxidative stress parameters in lungs following bleomycin administration. It was detected that bleomycin reduces the level of the activities of SOD, CAT, and GPx, as the main intracellular defense system against oxidative damage. Lipid peroxidation is known as one of the main consequences induced by oxidative stress, by which ROS bind to membrane lipids leading to their peroxidation followed by membrane disturbance and cellular damage. One of the important mediators produced during the process of membrane lipid peroxidation in MDA that show the severity of oxidative damage in cells [35]. However, our results demonstrated that bleomycin treatment significantly increased the level of MDA indicating pulmonary damage due to cellular injury.

The main purpose of this study was to evaluate and compare therapeutic potential of metformin and ascorbic acid against oxidative pulmonary fibrosis induced by bleomycin. This is noteworthy to indicate that both of metformin and ascorbic acid showed therapeutic potential when administrated alone in these experimental doses. Biochemical analyses showed that metformin and ascorbic acid increased the ability of cellular antioxidative defense system to combat oxidative stress, as there was a significant increase in the activities of SOD, GPx, and CAT. However, as mentioned, this is important to note that combination therapy with metformin and ascorbic acid has more efficacy in reducing the oxidative damage induced by bleomycin. Although previous studies examined the possible protective effects of metformin against bleomycin-induced pulmonary fibrosis [36, 37], the dose of metformin administrated is important due to its adverse effects on other organs [38]. It seems that one of the best ways to prevent this issue while receiving suitable therapeutic response is to combine metformin with other safe antioxidative agents such as ascorbic acid. However, there are a wide variety of results indicating the protective effects of metformin and ascorbic acid in different pathologies, that are in line with our results. For instance, it has been reported that metformin exhibits a protective role against paraquat induced lung injury via modulating the activity of SOD, GPx, and CAT followed by reduced MDA levels in lungs [39]. On the other hand, the same results have been observed due to treatment with ascorbic acid in different pathologies [40–42]. However, combination therapy with metformin and ascorbic acid has been considered in a couple of studies. For instance, it has been observed that co-treatment with metformin and ascorbic acid reduces type 2 diabetes mellitus and comorbid depression possibly via suppression of oxidative stress in rats [43]. Indeed, another study reveals that metformin and ascorbic acid provides better results in reducing the oxidative damage in diabetic myopathy in comparison with metformin alone [44]. Altogether, our results along with previous reports show that combination therapy with metformin and ascorbic acid has a suitable therapeutic potential against oxidative injury, especially pulmonary fibrosis induced by bleomycin. The main limitation of our study was the lack of information on other pathologic pathways involved in bleomycin induced pulmonary fibrosis, such as inflammatory responses and apoptosis.

## Figures and Tables

**Table 1:** Scores of inflammations and fibrosis of lung tissue in different groups in mice.

Groups	Control	Bleomycin	Bleomycin + metformin	Bleomycin + vitamin C	Bleomycin + methyl prednisolone	Bleomycin + metformin + vitamin C
Inflammation	1	4	3	3	2	2
Fibrosis	1	6	4	4	3	2

## Data Availability

Data will be available if requested.
